# Chlamydia psittacosis infection complicating cerebral infarction: A case report and literature review

**DOI:** 10.1097/MD.0000000000044854

**Published:** 2025-09-26

**Authors:** Jiadan Gao, Caili Lao, Jiangfeng Chen

**Affiliations:** a Department of Emergency, Shaoxing People’s Hospital, Shaoxing, Zhejiang, China; b The First Affiliated Hospital, Shaoxing University, Shaoxing, Zhejiang, China.

**Keywords:** cerebral infarction, *Chlamydia psittaci*, infection-associated thrombosis, metagenomic next-generation sequencing, severe pneumonia

## Abstract

**Rationale:**

*Chlamydia psittaci* (CP) pneumonia is a rare zoonosis. Severe infection predisposes patients to thrombotic complications; however, only 2 documented cases of delayed cerebral infarction occurring during anticoagulant therapy exist in the literature.

**Patient concerns:**

A 60-year-old female with a history of hypertension and a concealed history of avian exposure presented with fever, chest tightness, and dyspnea. Examination revealed severe hypoxemia (PaO_2_ 46.8 mm Hg), systemic inflammation (C-reactive protein 343.65 mg/L), and multi-organ dysfunction.

**Diagnoses:**

CP pneumonia; cerebral infarction.

**Interventions:**

Following definitive diagnosis via metagenomic next-generation sequencing of bronchoalveolar lavage fluid, targeted antimicrobial therapy with omadacycline (100 mg daily) and moxifloxacin (400 mg daily) was initiated immediately. Concurrent interventions included mechanical ventilation and prophylactic anticoagulation with enoxaparin (5000 IU daily). Upon development of an acute cerebral infarction, the antithrombotic strategy was modified: anticoagulation was discontinued and dual antiplatelet therapy (aspirin 100 mg/d + clopidogrel 75 mg/d) was commenced, alongside early rehabilitation in a dedicated stroke unit.

**Outcomes:**

By day 5 of antimicrobial therapy, inflammatory markers decreased significantly and oxygenation improved. Neurological function showed partial recovery by day 14 postinfarction (National Institutes of Health Stroke Scale score reduced from 8 to 3), enabling successful weaning from ventilation and hospital discharge.

**Lessons:**

Active screening for avian contact history and early application of metagenomic next-generation sequencing should be considered in patients with severe pneumonia. CP infection can trigger immunothrombosis, warranting vigilance for delayed stroke even during anticoagulation. Multidisciplinary management is crucial for optimizing outcomes in infection-associated cerebral infarction.

## 1. Introduction

Psittacosis, caused by the obligate intracellular bacterium *Chlamydia psittaci* (CP), is a zoonosis primarily transmitted by birds.^[1]^ Human infection occurs via inhalation of aerosolized avian secretions, presenting from self-limiting influenza-like illness to rapidly progressive severe pneumonia.^[2,3]^ Delayed diagnosis and deferred effective antibiotic therapy heighten mortality in severe cases. Conventional CP diagnostics remain limited: culture sensitivity is < 1%, and serology requires 2 to 3 weeks due to antibody maturation.^[4]^ Metagenomic next-generation sequencing (mNGS) now enables rapid, hypothesis-free pathogen detection without prior amplification.^[5]^ This case report details severe CP pneumonia with multi-organ dysfunction and acute cerebral infarction. mNGS of bronchoalveolar lavage fluid rapidly confirmed CP infection. Combined omadacycline therapy and stroke-unit management facilitated significant neurological recovery, highlighting critical benefits of integrated strategies for high-risk psittacosis.

## 2. Case presentation

**Figure 1.**
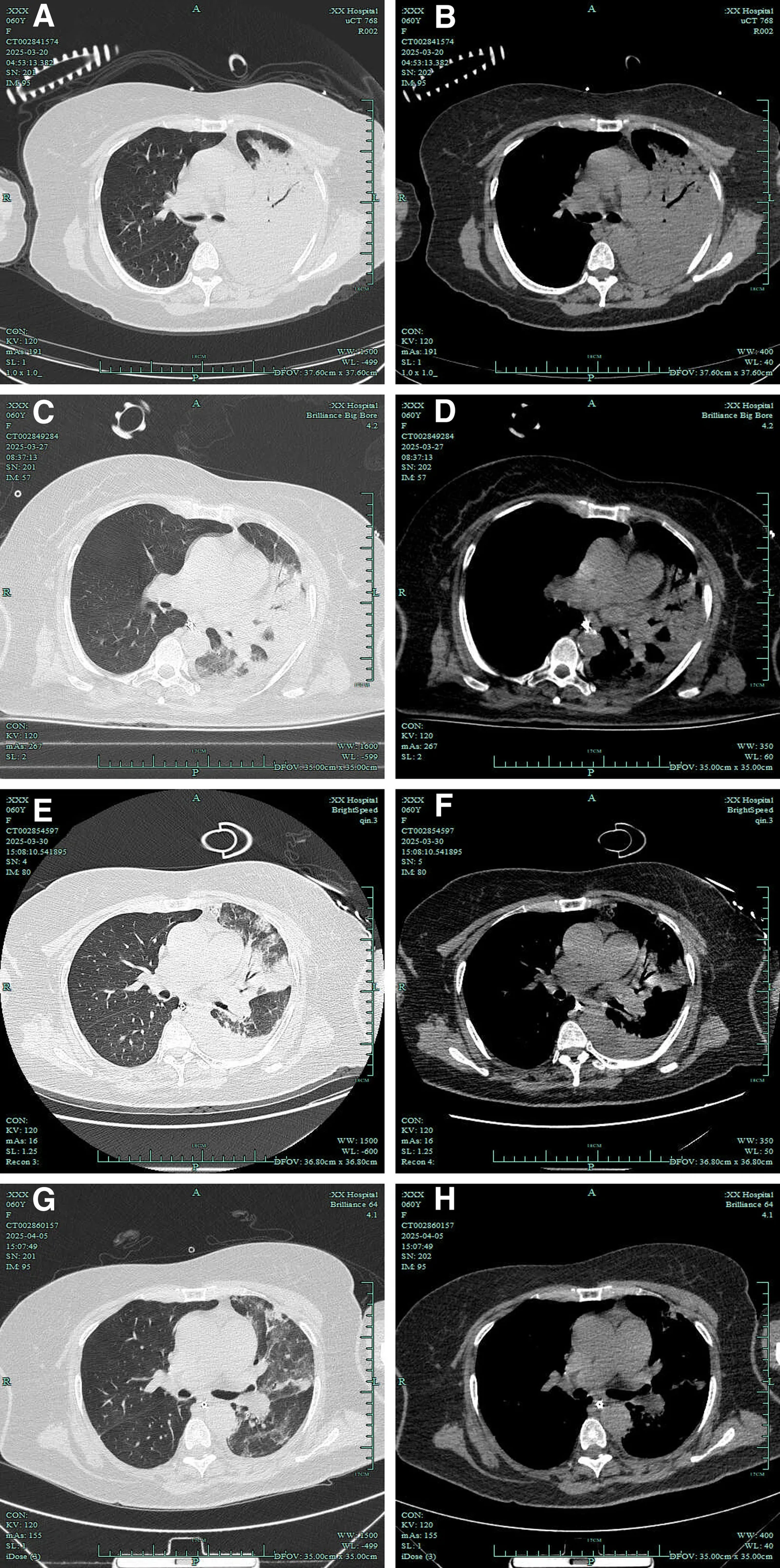
Chest CT images of a 60-year-old woman with severe *Chlamydia psittaci* pneumonia. (A, B) Chest CT on 20 March showed mostly solid left lung with a small amount of effusion; (C, D) Chest CT on 27 March showed most of the solid left lung better than before; (E, F) Chest CT on 30 March showed inflammation and absorption of the effusion compared with the previous one; (G, H) Chest CT on 5 April suggested exudation and solid left lung significantly better than before.

On March 20, 2025, a 60-year-old woman with a history of hypertension was admitted for progressive chest tightness, dyspnea, and fever lasting 1 week. Her initial symptoms included persistent high-grade fever with a peak temperature of 39.0°C, productive cough, nausea, and dizziness, and she had no chest pain or headache. Physical examination revealed tachycardia (91 bpm), tachypnea (26 breaths/min), hypoxemia (SpO_2_ 70% on room air), and moist rales in both lungs. Vital signs showed elevated blood pressure (173/92 mm Hg) and fever (38.2°C). Laboratory findings demonstrated systemic inflammation, including white blood cell count of 16.29 × 10^9^/L, C-reactive protein (CRP) of 343.65 mg/L, and procalcitonin of 5.63 ng/mL. Additionally, severe hypoxemia was noted with a PH of 7.457, PaCO_2_ of 45.1 mm Hg, and PaO_2_ of 46.8 mm Hg. Multi-organ injury was also evident, with alanine aminotransferase/aspartate aminotransferase (ALT/AST) levels of 145.4/193.6 U/L and creatine kinase/creatine kinase-MB levels of 829.1/831.3 U/L (see Table 1). Thrombotic screening revealed markedly elevated d-dimer (22.31 mg/L), and lower extremity ultrasonography identified a left calf muscular vein thrombosis. Scanning computed tomography (CT) of the head showed old lesions in the right lateral paraventricular and basal ganglia regions. Enhanced CT of the chest revealed mostly solid lesions in the left lung and a small amount of effusion in the left pleural cavity (Fig. 1A, B). The patient underwent immediate bedside endotracheal intubation in the emergency department for mechanical ventilation and was transferred to the intensive care unit. Empirical antibiotic therapy with piperacillin–sulbactam (5.0 g, q8h) and moxifloxacin (0.4 g, qd) was initiated. Given the severity of presentation and suspicion of atypical pathogen involvement, bronchoalveolar lavage (BAL) was performed on admission day; BAL fluid was subjected to mNGS. Forty-eight hours later, mNGS confirmed CP infection. Retrospective history-taking revealed exposure to a domestic rooster. The diagnosis was revised to severe psittacosis pneumonia complicated by respiratory failure, hepatic dysfunction, electrolyte imbalance, and venous thromboembolism. Antimicrobial therapy was escalated to intravenous omadacycline (0.1 g, qd; loading dose 0.2 g) and moxifloxacin (0.4 g, qd). Adjunctive therapies included prone positioning ventilation, glutathione (1.2 g qd) for hepatoprotection, low-molecular-weight heparin (5000 IU qd), volume management, sedation and analgesia, and temperature management.

**Table 1:** Laboratory indicators of the patients.

Parameters	Normal range	The day of admission	Predischarge
White blood cell count (×10^9^/L)	3.5–9.5	16.29	9.85
Neutrophil count (×10^9^/L)	1.8–6.3	14.31	7.91
Neutrophil (%)	40–75	87.9	80.3
Lymphocyte count (×10^9^/L)	1.1–3.2	1.08	1.26
Lymphocyte (%)	20–50	6.6	12.8
Platelets (×10^9^/L)	125–350	310	342.0
CRP (mg/L)	0–6	343.65	4.3
PCT (ng/mL)	0–0.5	5.63	0.18
d-Dimer (mg/L)	0–0.7	22.31	1.91
ALT (U/L)	7–40	145.4	78.6
AST (U/L)	13–35	193.6	27.9
Na^+^ (mmol/L)	137–147	127.8	141.7
Cl^‐^ (mmol/L)	99–110	84.3	99.5
CK (U/L)	26–140	829.1	24.4
CK-MB (U/L)	0–25	31.3	10.5
TPI (ng/mL)	0–0.026	0.039	0.004

ALT = alanine aminotransferase, AST = aspartate aminotransferase, CK = creatine kinase, CK-MB = creatine kinase-MB, Cl = chloride, CRP = C-reactive protein, Na = sodium, PCT = procalcitonin, TPI = troponin I.

By hospital day 3, the patient exhibited reduced fever spikes and improved oxygenation, enabling gradual downward adjustment of ventilator parameters. Repeat chest CT on March 27 showed large solid lesions of the left lung with left-sided pleural effusion (Fig. 1C, D). However, on the 10th day of admission (March 30, 2025), the patient presented with right limb weakness, right limb muscle strength grade 0 (Medical Research Council scale), normal muscle tone, left limb muscle strength grade 5, and a National Institutes of Health Stroke Scale score of 8. A cranial CT examination suggested abnormalities adjacent to the right lateral ventricle and bilateral basal ganglia. A chest CT revealed a solid lesion in the left lung with a left-sided pleural effusion (Fig. 1E and F). The stroke team was asked to recommend that a cranial magnetic resonance imaging (MRI) be performed for further clarification.

**Figure 2.**
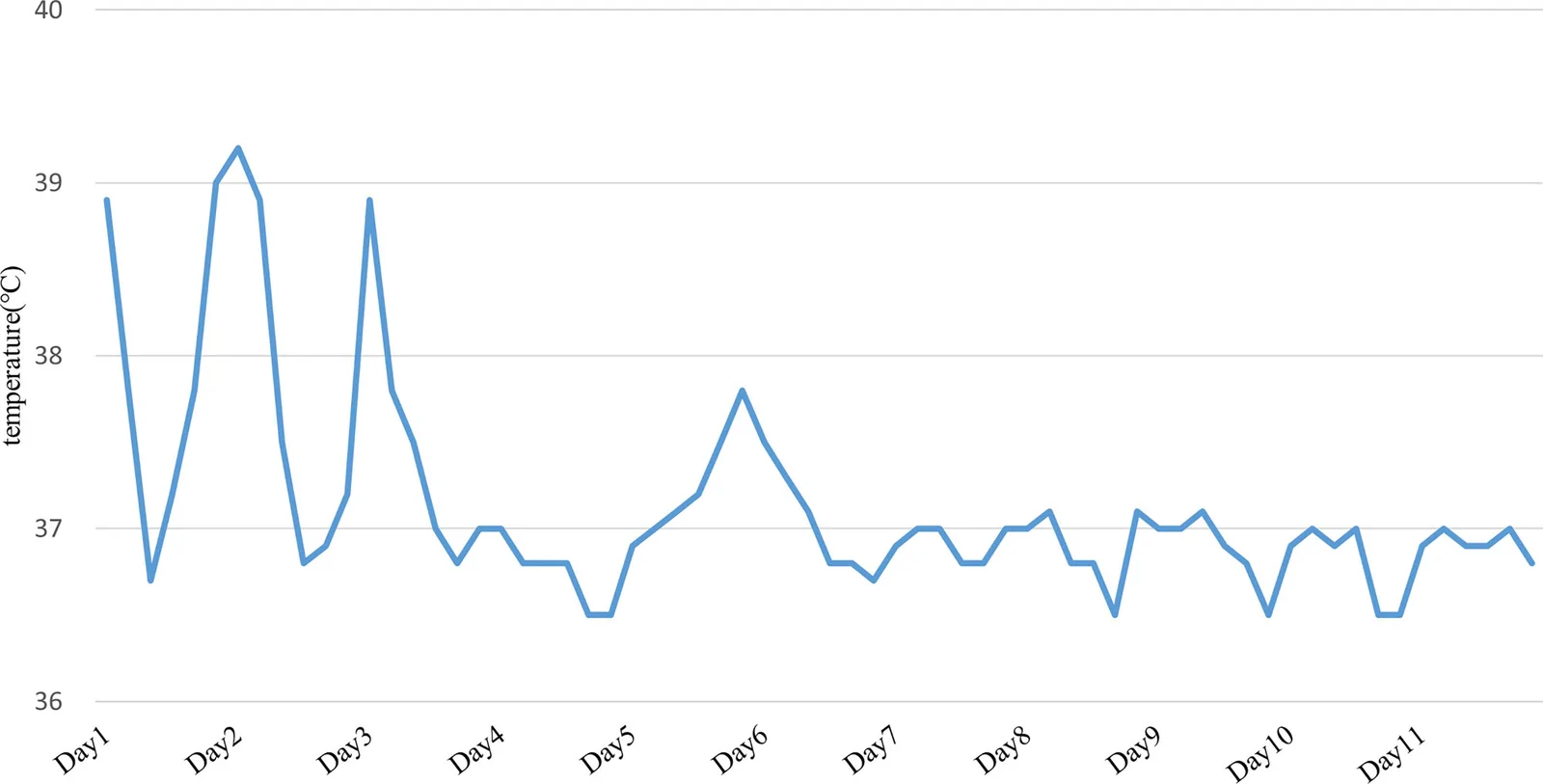
Trend of patient’s temperature during hospitalization.

**Figure 3.**
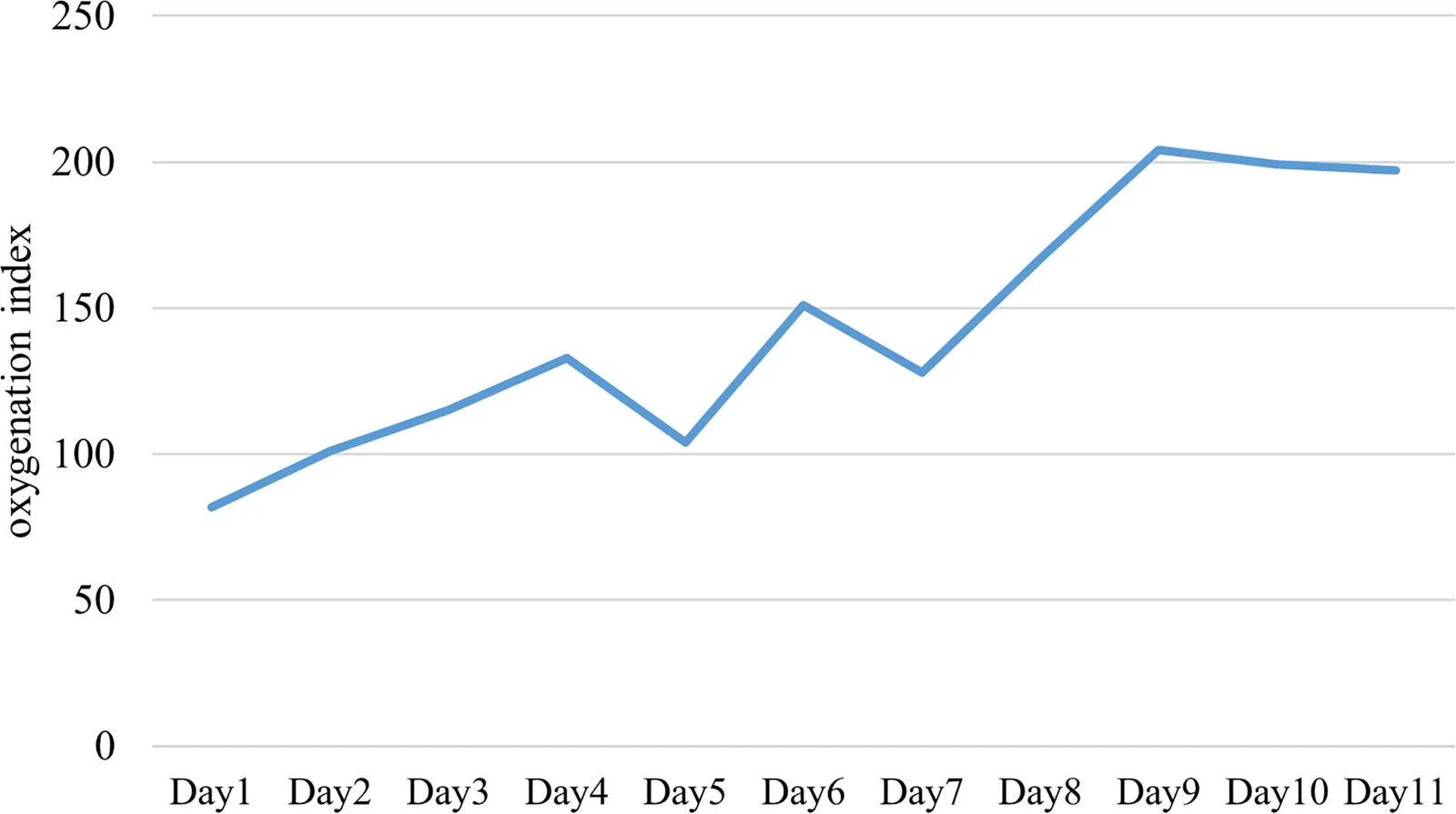
Graph of oxygenation index during the patient’s hospitalization.

**Figure 4.**
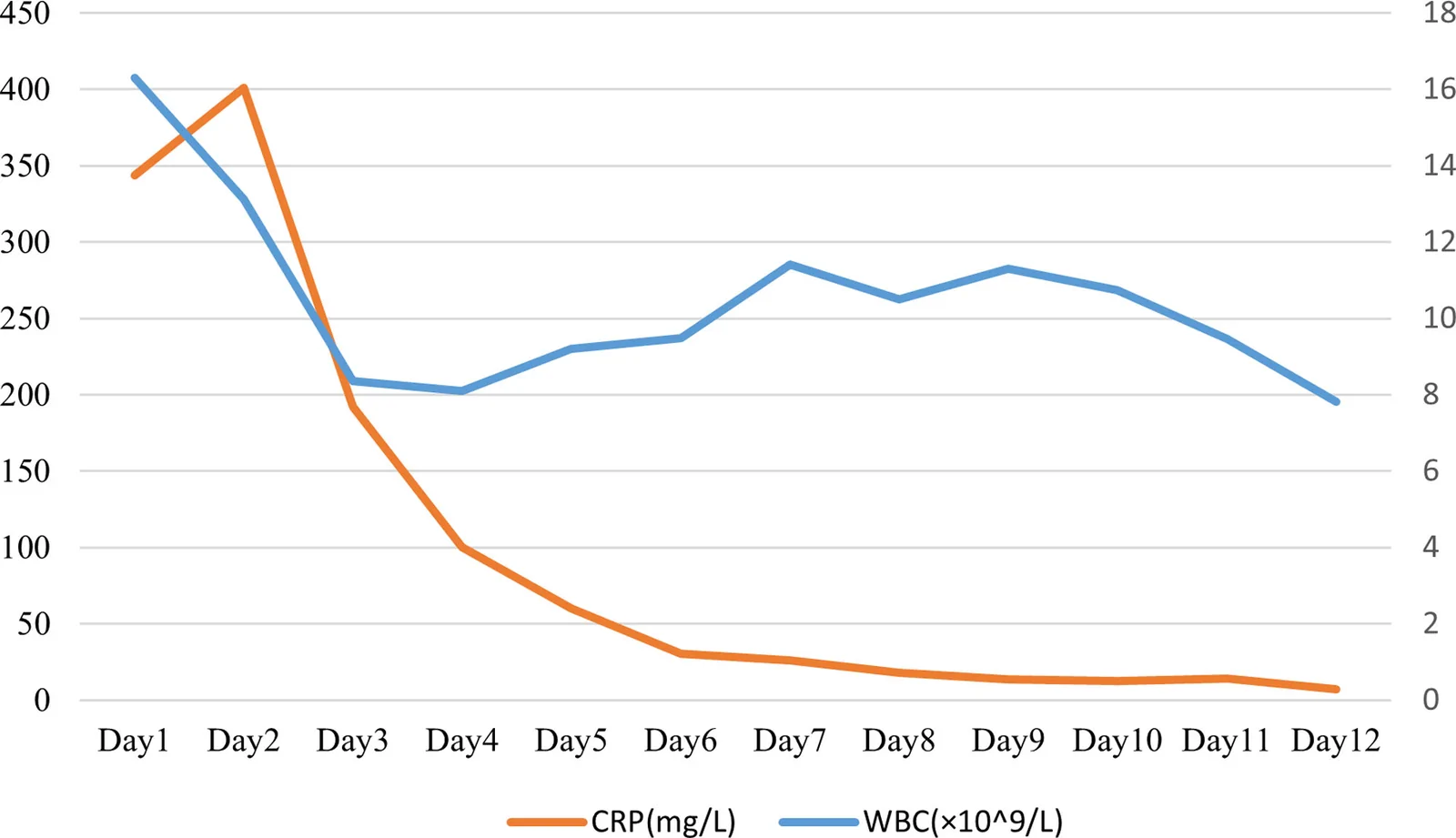
Changes in WBC and CRP during hospitalization. CRP = C-reactive protein, WBC = white blood cell.

As the patient’s temperature gradually subsided (Fig. 2), oxygen saturation and other relevant indices increased (Fig. 3), and inflammatory markers, including white blood cell and CRP, continued to decrease (Fig. 4). The antibiotic regimen was stepped down to doxycycline tablets 0.1 g orally every 12 hours. A detailed cranial MRI was performed on April 1 after extubation of tracheal intubation, revealing multiple small new infarcts in the posterior branch of the left internal capsule, thalamus, and posterior part of the corona radiata. The patient was then transferred to the Department of Neurology for treatment. Low molecular weight heparin was discontinued, and treatment with dual antiplatelet therapy (aspirin 100 mg plus clopidogrel 75 mg), butylphthalide injection to improve cerebral circulation, edaravone and dexamethasone to scavenge oxygen free radicals, as well as rehabilitation therapy, were initiated. During follow-up, chest CT showed that the infected lesion in the left lung was accompanied by a small amount of effusion (Fig. 1G and H). Carotid artery CTA demonstrated severe stenosis of the M1 segment of the right middle cerebral artery; however, this diseased vessel had no blood flow supply relationship with the infarcted area. Dynamic electrocardiogram showed sinus rhythm. There were no abnormalities in the metabolism of antinuclear antibody, antiphospholipid antibody, homocysteine, or lipids. Subsequently, the patient’s muscle strength gradually improved. By April 8, the muscle strength of the right upper limb was grade 3, and that of the right lower limb was grade 4. There was no choking on drinking water. The patient was discharged for further rehabilitation training.

## 3. Discussion

This case presents a complex clinical course of CP pneumonia complicated by multi-organ dysfunction and delayed cerebral infarction. Its management involves the treatment of respiratory critical illness, innovations in microbiological testing techniques, and exploration of pathomechanisms underlying infection-associated thrombotic events; this is an important clinical insight.

### 3.1. Challenges in the management of Chlamydia pneumonia in psittacosis

CP, an obligate intracellular pathogen, exhibits marked variability in infection latency, with a median incubation period of 5 to 14 days and documented extremes up to 39 days. The disease manifests with highly heterogeneous clinical presentations: classic features include acute-onset high fever, severe headache, and nonproductive cough, though asymptomatic carriage has been reported.^[6]^ Common systemic symptoms encompass fatigue, dyspnea, chills, and myalgia.^[7]^ Notably, CP infection may rarely present with pathognomonic signs such as pulse-temperature dissociation (Faget sign), splenomegaly, or erythematous rash. Moreover, severe CP infections often begin as atypical pneumonia with rapidly progressive multilobar solid lesions and bronchial inflation signs on chest imaging. While neurological complications remain uncommon, they demonstrate broad phenotypic diversity, ranging from encephalitis to cerebellar dysfunction, transverse myelitis, intracranial hypertension, and cranial neuropathies.^[8]^

The present case exhibited fever, left lung infiltration, and multi-organ injury, which are hallmarks of severe CP pneumonia. However, the initially unclear epidemiological link (later traced to domestic rooster exposure) underscores the indispensable need for meticulous history-taking in atypical presentations.

Conventional culture methods demonstrate limited utility for CP detection. PCR-based direct pathogen detection reduces turnaround time but requires prior clinical suspicion to select target pathogens.^[9]^ A Chinese multicenter retrospective study revealed that CP accounts for 6.8% of severe community-acquired pneumonia cases.^[10]^ The advent of mNGS has addressed these limitations,^[11]^ enabling atypical pathogen identification within 36 to 48 hours and facilitating early targeted antimicrobial therapy for improved outcomes.^[12]^ Current consensus considers CP clinically significant when detected via mNGS in bronchoalveolar lavage fluid, given its high specificity in lower respiratory specimens.^[13]^ In this case, BAL fluid-mNGS confirmed CP infection within 48 hours of admission, crucially guiding pathogen-directed therapy with omadacycline.

### 3.2. Optimization of anti-infective treatment strategies

CP demonstrates preserved susceptibility to tetracyclines, macrolides, and fluoroquinolones, with tetracyclines remaining the first-line therapy according to international guidelines for CP pneumonia.^[14]^ This case initially received empirical piperacillin–tazobactam (5.0 g every 8 hours) combined with moxifloxacin (0.4 g once daily). However, persistent fever and escalating inflammatory markers (CRP: 343.65–398.12 mg/L; procalcitonin: 5.63–8.91 ng/mL) at 72 hours led to antimicrobial escalation. Treatment was switched to omadacycline (0.1 g once daily, with a 0.2 g loading dose) plus moxifloxacin, guided by mNGS-confirmed CP detection. This adjustment was made to improve therapeutic efficacy. Omadacycline, a third-generation tetracycline derivative approved by the U.S. Food and Drug Administration in October 2018 for community-acquired bacterial pneumonia and acute bacterial skin and skin structure infections,^[15]^ exhibits structural advantages over earlier tetracyclines. Its alkylaminomethyl modification at the C-9 position of the d-ring—replacing the glycinamide group found in minocycline—confers resistance to common tetracycline efflux pumps and ribosomal protection mechanisms.^[16]^ Pharmacokinetically, omadacycline achieves lung concentrations that exceed plasma levels by 2.5-fold. Additionally, it is primarily eliminated via feces, which eliminates the need for dose adjustments in patients with hepatic or renal impairment_._^[17]^ This profile contrasts sharply with that of older tetracyclines, such as doxycycline and minocycline, which carry well-documented risks of drug-induced hepatotoxicity.^[18]^

### 3.3. Inflammatory response and thrombotic events

Stroke imposes substantial global socioeconomic burdens, accounting for $721 billion (0.66% of global GDP) in 2022.^[19]^ In our case, markedly elevated d-dimer (peak: 22.31 mg/L; ref. <0.5 mg/L) coincided with deep vein thrombosis and delayed cerebral infarction, implicating infection-associated immunothrombosis. CP infection triggers systemic inflammation via macrophage hyperactivation, dysregulated cytokine release (e.g., interleukin [IL]-6, TNF-α, and IL-10), and TLR4/Mal/MyD88/NF-κB signaling. Concurrently, it impairs endothelial function—typically responsible for anticoagulant, vasodilatory, and antiplatelet regulation—through cytokine-driven activation (e.g., TNF-α, IFN-γ).^[20–22]^ This endothelial dysfunction shifts vascular homeostasis toward vasoconstriction, increased permeability, and a hypercoagulable state, culminating in thrombosis during inflammatory stress.^[23]^

### 3.4. Exploring the mechanisms of neurovascular events

The patient developed acute right-sided hemiplegia on hospital day 10, with MRI confirming a fresh infarct in the left posterior limb of the internal capsule. The pathogenesis may involve multiple CP-specific mechanisms:

Hematogenous dissemination: Following respiratory entry, CP undergoes rapid replication and systemic spread through the bloodstream, with involvement of the reticuloendothelial system, inducing vasculotropic effects in neural tissues.^[24]^

Molecular mimicry: Previous studies have shown that certain proteins in CP have sequences similar to peripheral nerve tissue proteins. CP infection leads to the production of autoantibodies, which can damage peripheral nerves, spinal cord nerves, and central nervous system nerves as a result of antigen–antibody interactions leading to peripheral nerve complement-mediated nerve injury.^[25]^

IL-2 mediated neuroinflammation: Additionally, psittacosis has been reported to promote the release of inflammatory cytokines, including IL-2, a multifunctional molecule produced by multiple cell types. IL-2 has been implicated in modulating susceptibility and progression in immune-mediated central nervous system disorders such as ischemic stroke.^[26]^

Notably, comprehensive workup excluded embolic sources (infective endocarditis, atrial fibrillation), atherosclerotic plaque rupture, and antiphospholipid syndrome.

### 3.5. The key role of multidisciplinary treatment

The successful treatment of this case highlights the advantages of the multidisciplinary treatment model: First, early mechanical ventilation combined with prone positioning by the critical care team improved oxygenation, as evidenced by an increase in the PaO_2_ from 89 mm Hg at admission to 197 mm Hg at extubation, consistent with the lung-protective ventilation strategy; Second, the adjustment of the anticoagulation regimen reflected a balance between thrombosis and hemorrhage: initially, low-molecular-weight heparin (enoxaparin 5000 IU qd) was used to prevent venous thromboembolism, followed by a switch to dual antiplatelet therapy (aspirin + clopidogrel) after the infarction, in accordance with the 2024 Stroke guideline recommendations for secondary prevention of infection-associated cerebral infarction^[27]^; Third, early bedside rehabilitation intervention was initiated within 72 hours of symptom onset to facilitate neurological recovery, resulting in a decrease of the National Institutes of Health Stroke Scale score from 12 to 4. These measures collectively contributed to the patient’s improved prognosis.

### 3.6. Characteristics of liver injury in CP pneumonia

CP initiates infection via respiratory entry, subsequently replicating within reticuloendothelial cells of the liver and spleen before hematogenous dissemination to pulmonary and extrapulmonary sites. The rising incidence of reported CP pneumonia cases has paralleled increased recognition of its multisystemic manifestations, with hepatic involvement emerging as a cardinal feature. Patients with CP pneumonia have been shown to exhibit elevated levels of ALT and AST.^[28]^ A retrospective analysis by Li et al (n = 52) demonstrated AST and ALT elevation in 86.5% and 75.0% of cases, respectively.^[29]^ Similarly, Su et al identified significantly higher AST and lactate dehydrogenase levels in severe CP pneumonia cohorts.^[30]^ The presence of Chlamydia species predominantly in peripheral blood mononuclear cells from different organs, including the liver, may help to amplify the local and systemic inflammatory response against Chlamydia infection. This inflammatory response promotes abnormal liver function and the production of CRP and d-dimer, which partly explains the liver damage in infected patients.^[21]^

### 3.7. Evolutionary features of imaging dynamics

Serial chest CT showed a typical course of the left lung from a large solid lesion (day 1) to partial resorption (day 21). The progression can be divided into 3 phases: acute phase (day 1–7), characterized by fibrin exudation in the alveolar lumen with hyaline membrane formation, corresponding to a CT ground-glass shadow; progressive phase (day 8–14), marked by macrophage infiltration leading to a solid lesion, with bronchial dilatation visible; and resorption phase (day 15 and beyond), featuring fibroblast proliferation with interstitial remodeling, leaving behind fibrotic stripes. It is worth noting that the phenomenon of “image-symptom separation” occurs in about 15% of patients. In these cases, radiological improvement lags behind clinical symptom improvement by 2 to 3 weeks.

### 3.8. Public health significance

Clinical and environmental recognition of CP is underestimated worldwide; therefore, the true disease burden remains unknown. A meta-analysis in 2021 described the overall prevalence of chlamydial infection in birds as 20% globally, and the prevalence has been relatively consistent since 2012.^[31]^ The domesticated rooster owned by our patient served as a source of infection, highlighting the need for improved health surveillance of poultry farmers. Although studies have shown a CP antibody positivity rate of 8.3% among live poultry market workers, there is currently no statutory reporting system for Psittacosis in China. It is recommended to refer to the European Union case definition criteria, which include clinical pneumonia, epidemiological exposure, and laboratory confirmation of diagnosis. Additionally, it is advised to establish a zoonotic disease surveillance network.

## 4. Conclusion

This rare case of severe CP pneumonia with concomitant lower-extremity venous thrombosis and delayed cerebral infarction underscores the pathogen’s multisystem invasiveness and complex thromboembolic risk profile. Metagenomic next-generation sequencing of bronchoalveolar lavage fluid rapidly confirmed cryptic avian exposure, demonstrating the critical role of molecular diagnostics in pathogen identification. Integrated multidisciplinary management (prone-position ventilation, imaging-guided antithrombotic adjustment, and early rehabilitation) was pivotal for multisystem recovery.

## Acknowledgments

We thank the patient and her family for giving us permission to share this rare case, and express our gratitude to the imaging department for providing us with imaging data.

## Author contributions

**Writing – original draft:** Jiadan Gao.

**Writing – review & editing:** Caili Lao, Jiangfeng Chen.
